# Streamlining Culture Conditions for the Neuroblastoma Cell Line SH-SY5Y: A Prerequisite for Functional Studies

**DOI:** 10.3390/mps5040058

**Published:** 2022-07-12

**Authors:** Sebastian Feles, Christian Overath, Sina Reichardt, Sebastian Diegeler, Claudia Schmitz, Jessica Kronenberg, Christa Baumstark-Khan, Ruth Hemmersbach, Christine E. Hellweg, Christian Liemersdorf

**Affiliations:** 1 Radiation Biology, Institute of Aerospace Medicine, German Aerospace Center (DLR), 51147 Cologne, Germany; sebastian.diegeler@utsouthwestern.edu (S.D.); claudia.schmitz@dlr.de (C.S.); jessica.kronenberg@dlr.de (J.K.); christa.baumstark-khan@dlr.de (C.B.-K.); christine.hellweg@dlr.de (C.E.H.); 2 Gravitational Biology, Institute of Aerospace Medicine, German Aerospace Center (DLR), 51147 Cologne, Germany; ruth.hemmersbach@dlr.de (R.H.); christian.liemersdorf@dlr.de (C.L.); 3 Institute of Virology, University Hospital of Cologne, 50935 Cologne, Germany; christian.overath@uk-koeln.de; 4 Biofrontera Bioscience GmbH, 51377 Leverkusen, Germany; s.reichardt@biofrontera.com

**Keywords:** neuroscience, *in vitro* methods, SH-SY5Y, proliferation, characterization, neuroblastoma, morphology, cultivation techniques, cell culture

## Abstract

The neuroblastoma cell line SH-SY5Y has been a well-established and very popular *in vitro* model in neuroscience for decades, especially focusing on neurodevelopmental disorders, such as Parkinson’s disease. The ability of this cell type to differentiate compared with other models in neurobiology makes it one of the few suitable models without having to rely on a primary culture of neuronal cells. Over the years, various, partly contradictory, methods of cultivation have been reported. This study is intended to provide a comprehensive guide to the *in vitro* cultivation of undifferentiated SH-SY5Y cells. For this purpose, the morphology of the cell line and the differentiation of the individual subtypes are described, and instructions for cell culture practice and long-term cryoconservation are provided. We describe the key growth characteristics of this cell line, including proliferation and confluency data, optimal initial seeding cell numbers, and a comparison of different culture media and cell viability during cultivation. Furthermore, applying an optimized protocol in a long-term cultivation over 60 days, we show that cumulative population doubling (CPD) is constant over time and does not decrease with incremental passage, enabling stable cultivation, for example, for recurrent differentiation to achieve the highest possible reproducibility in subsequent analyses. Therefore, we provide a solid guidance for future research that employs the neuroblastoma cell line SH-SY5Y.

## 1. Introduction

The human neuroblastoma cell line SH-SY5Y has been widely used as an *in vitro* model for a variety of neurobiological analyses for several decades [1,2]. Nevertheless, detailed information on its cultivation and cell biological characterization is scarce, and the literature is incoherent on these points. Basic proliferation parameters seem to have never been fully captured. Only single parameters are mentioned in the literature (DSMZ (Braunschweig, Germany): ACC 209; Sigma-Aldrich (St. Louis, MO, USA): 94030304, ATCC (Manassas, VA, USA): CRL-2266) [3]. Other information is distributed over many publications and has to be collected laboriously. This study is intended to serve as an instruction manual for the establishment and use of the SH-SY5Y cell line in the steady-state and undifferentiated form. These cells can be further differentiated into a neuronlike state, in which projections resembling neurites are formed. The differentiation process can be induced in several various ways (e.g., by stimulating with retinoic acid) [2].

The cancer cell line SH-SY5Y is a subclone of the primary neuroblast-like cell line SK-N-SH, which originates from a bone marrow biopsy containing metastatic neuroblastoma tissue in 1973 [4]. Biedler et al. derived the subclone SH-SY from the SK-N-SH cell line, which in turn was passaged to the subclone SH-SY5 [4]. Further subcloning of SH-SY5 created the still widely employed SH-SY5Y neuroblastoma cell line [5,6]. SH-SY5Y cells are a popular *in vitro* model for the investigation of neuronal function and differentiation and are used, for example, for the investigation of neurodevelopmental disorders, such as Parkinson’s disease or neurogenesis [7,8]. The cell line is particularly suitable for research in neurodegenerative diseases due to the fact that it has a specific uptake of the neurotransmitter norepinephrine (NA), expresses bona fide neurofilament proteins, and shows activity of well-known enzymes involved in catecholaminergic systems (tyrosine- and dopamine-β-hydroxylases) [9,10,11]. In addition, the cells express opioid, muscarinic, and NGF (nerve growth factor) receptors [12]. Thus, SH-SY5Y cells are used especially for research focusing on the adrenergic and/or dopaminergic systems [13,14]. In addition to Parkinson’s research, recent studies with this versatile cell line are concerned with neuronal characterization following ultrasound stimulation [15] or the formation of human neuroblastoma in mouse–human neural crest chimeras [16]. Since the introduction of the cell line in 1978, it has gone through many passages that indicate sufficient chromosome stability [17]. The results shown in our study should therefore be comparable from laboratory to laboratory. Similarly, to many immortalized tumor-tissue-derived cell lines, SH-SY5Y cells have the advantage of indefinite cell expansion comparable to many immortalized cell lines. The disadvantage is an undefined differentiation state, ranging from tumor tissue state (neuroblastoma) to neural progenitor cells or postmitotic neurons. Therefore, SH-SY5Y cells are considered to show a “neuronlike” phenotype upon induced differentiation into the mature neuron-like state. While the outgrowth of neuronal processes is studied frequently in differentiated SH-SY5Y cells [1,2,18], mechanisms such as neuronal function and excitability cannot be readily compared with, for instance, a physiological state of brain-tissue-derived cells.

The culture of differentiated cells is complex and time-consuming. After entry into the G_0_ phase, differentiated SH-SY5Y cells do not proliferate. This excludes all cell biology experiments, such as cell cycle analysis or growth curves that rely on cell proliferation. Here, we describe the stable long-term cultivation of undifferentiated SH-SY5Y that can be used, for example, for several differentiation paradigms derived from the same stock culture, thus achieving a very high reproducibility. A complete and comprehensive explanation of procedures for the differentiation and separation of the SH-SY5Y cells has been published [1,2]. In these studies, the 18-day process for differentiation and isolation of differentiated SH-SY5Y using retinoic acid was described in detail, but details on the cultivation of the undifferentiated cells are missing. The prerequisite of any differentiated cell culture is a well-established and standardized culture of undifferentiated cells. Our study is the first, to our knowledge, to provide details for exactly this purpose.

In this study, we provide the general cultivation procedure and growth parameters, such as proliferation rates and robust instructions, which ensures a high degree of differentiation efficiency, for successful experiments with this cell line.

## 2. Materials and Methods

### 2.1. Cultivation of SH-SY5Y Cells

The SH-SY5Y cells were obtained from Prof. Dr. Thorsten Lang (Limes Institute, University of Bonn, Germany) via ATCC (CRL-2266) and were cultured in a passage lower than 100 from the original stock in a 1:1 mix of DMEM (Pan Biotech, Aidenbach, Germany) #P04-03600, Lot No. 4181014) and Ham’s F12 (Pan Biotech #P04-15500, Lot No. 5400217) completed with 1% *v*/*v* L-glutamine (Pan Biotech #P04-80100, Lot No. 4540117), 1% *v*/*v* penicillin–streptomycin (Pan Biotech #P06-07100, Lot No. 4281216), and 10% *v*/*v* FBS (#S0115, Lot No. 0740B, Biochrom, Berlin, Germany), called NeuroMedium. DMEM was completed with 10% *v*/*v* FBS, 5% *v*/*v* L-glutamine [19], and 1% *v*/*v* penicillin–streptomycin. The SH-SY5Y cell line was authenticated by the control of key cellular parameters, such as outgrowth velocity upon initial seeding employing the same seeding density, general cell morphology, and spreading and adhesion behavior, as well as by retinoic-acid-induced differentiation. For a working environment, a spatially separated laboratory and solutions dedicated only to this cell line were used, and therefore, a contamination with another cell line, since the purchase from ATCC, can be excluded.

To ensure comparability between cultures, cell densities were measured before any experimental approach. Cell densities were measured using automated imaging and cell counting by a Luna cell counter (Logos Biosystems, Anyang, Korea). In order to distinguish living from dead cells, the cell suspension was mixed at a ratio of 1:1 with a 0.4% (*v*/*v*) trypan blue staining solution (Sigma-Aldrich: T8154). The stained cell suspension was transferred to disposable slides (Logos Biosystems, Anyang, Korea), in which stained and unstained cells were automatically counted. In addition to the cell number, the cell size distribution and the viability were obtained. Cell viability was determined based on trypan blue staining, which indicates disturbed cell membrane integrity in case of nonexclusion of the dye. Only adherently growing cells were used for cell counting and determination of cell viability. Cells in suspension were discarded before measurement. The importance of the free-floating cell population and its proportion in the culture is inconsistently described in the literature. Most of the publications attribute only a subordinate role to the cells in suspension and neglect their proportion in the total population. There are also recent studies that make vague assumptions that the free-floating cells could play a greater role than is generally assumed. Here, however, the data are still insufficient [1]. For this reason, the focus was placed only on adherent cells in this study.

For long-term storage, even over decades, cells were frozen and stored in liquid nitrogen tanks [1,2,20]. For the preparation of frozen cell stocks, cells were seeded in 75 cm^2^ culture flasks (Labsolute, Renningen, Germany) #7.696 782) with a cell density of 5.0 × 10^4^ cells/cm^2^. When the cells reached the culturing density of 80% confluence (approximately 5.0 × 10^5^ cells/cm^2^) after 7–8 days of incubation, they were collected for the freezing procedure. The medium was removed, the cells were washed with PBS and detached from the culture surface by 3 min incubation with a trypsin/EDTA solution (0.05%/0.02%, Pan Biotech #P10-024100, Lot No. 3130316). Subsequently, the enzymatic reaction was stopped using a culture medium containing 10% FBS, and the cell number was determined. The cell suspension was pelleted at 500× *g* for 5 min at room temperature, and the supernatant discarded. The cell pellet was resuspended in a cryostorage medium consisting of NeuroMedium + 10% DMSO at a cell concentration of 4.0 × 10^6^ cells/mL. Afterwards, the method followed the optimized protocol of deliberate cooling from 37 to −80 °C at a rate of −1 °C/min [21]. After 48 h, the cryotubes were transferred to liquid nitrogen.

When new batches of cells were needed, stock aliquots were thawed and cultivated further. For the thawing procedure, the cryotubes were removed from the liquid nitrogen. We recommend thawing at 37 °C according to the guidelines of commonly used protocols for SH-SY5Y cultures [1]. The cryostorage medium of the thawed cell suspension was added to fresh NeuroMedium at 4 °C in a standard 25 cm^2^ cell culture flask (Labsolute #7.696 781). We determined an optimal ratio of 1 part cryostorage medium to 5 parts fresh NeuroMedium for a cell density of 1.6 × 10^5^ cells/cm^2^. At this ratio, the DMSO concentration was 1.67% and thus slightly toxic for the cells over longer incubation periods [22]. For this reason, it is necessary to exchange the medium again after 24 h [23]. As an alternative, the DMSO can be removed by centrifugation before seeding.

SH-SY5Y cells were successfully cultured in standard cell culture pretreated polystyrene culture vessels at 37 °C, 5% CO_2_, and saturated humidity in an incubator [24,25]. A special coating of the growth surface is not necessary for the cultivation of undifferentiated adherent cells. A coating with 0.01 mg/mL poly-d-lysine is possible, if required for the experimental conditions, and has no negative impact on growth rate or morphology [26]. The cell line could be cultivated on untreated and noncoated glass without negative impacts as well. In differentiated cells, the coating might have a considerable influence on the morphology, such as the increase in neurite extensions [19].

Due to high metabolic consumption and rapidly accumulating metabolites, the exchange of medium was necessary at least every 3 days. The maximum duration of experiments was limited to 17 days, as cell viability was reduced to 85.5 ± 5.4% after about 14 days. During the culture period, there were initially less pronounced and later more intense cyclical fluctuations in viability due to the medium change cycle. Viability increases for a short time after a medium change and then decreases again until the next change. This effect is most distinctive at the end of the culture period and at complete confluence. Thus, after 17 days of cultivation, experimental results could no longer be clearly separated from influences of the accumulating metabolic waste products. Subsequent cultivation showed a rapid decrease in the pH value to a critical level after medium change so that constant pH levels could only be maintained by daily media changes. The resulting loss of growth and signaling factors in the media further influenced the proliferation and differentiation capacity, rendering these cells no more suitable for reliable experiments.

Contamination tests for *Mycoplasma* were performed annually by sending 1.5 mL cell culture supernatants to the German Collection of Microorganisms and Cell Cultures (DSMZ) for PCR-based detection of *Mycoplasma* using a primer mixture for the amplification of a conserved 16S rDNA region occurring in *Mycoplasma* species predominantly found in cell cultures and most of the other *Mycoplasma* species (including Myco-, Achole-, Urea-, and Spiroplasma). In none of the SH-SY5Y cultures *Mycoplasma* were detected.

### 2.2. Expansion of SH-SY5Y Cells

In order to expand the cell number, SH-SY5Y cells can be repeatedly detached and reattached to the culture vessel, and further growth to confluence was feasible. Successful splitting of the cells was possible according to standard protocol with trypsin/EDTA and fast detachment of the cells [27]. Optimal growth rates were achieved by passaging the cells at a confluence of ~80% because the cells were still in the exponential growth phase and present at high numbers without reaching the critical lag phase. Following the removal of the medium, the cells were washed once with PBS and subsequently detached with trypsin/EDTA as described previously. SH-SY5Y cells showed substantial adhesion to the culture surface; thus, enzymatic detachment was accelerated by incubating at 37 °C for a maximum of 5 min, avoiding cytotoxic effects for the cells [28]. The trypsin reaction was stopped with NeuroMedium containing 10% FBS at a ratio of 1:5. To remove the trypsin/EDTA solution, the cell suspension was centrifuged for 5 min at 500× *g*. The supernatant was discarded, and the pellet was resuspended in fresh NeuroMedium. The cell suspension obtained during this procedure should be homogeneously distributed by gently pipetting up and down repeatedly to avoid SH-SY5Y cells adhering to each other and forming cell clumps.

Different initial cell numbers were used to determine the plating efficiency (PE) and, thus, the colony-forming ability of SH-SY5Y. A total of 100, 200, 500, 1000, and 2000 cells/Petri dish (Ø 6 cm) were sown to six replicates each and incubated under standard conditions for 21 days. A colony was defined as ≥50 cells. The colony growth was monitored macroscopically, and colonies were stained for counting after each determined incubation time. For this purpose, the cells were washed with tap water and then stained with crystal violet 0.1% (*w/v*) in a 3.7% formaldehyde solution at room temperature. The quotient of the determined colony count per Petri dish and the seeded cell count describes the PE.

### 2.3. Data Acquisition and Statistical Analysis

The single cell area was calculated from values derived from acquired images. The cell number was determined by a Luna cell counter (Logos Biosystems, Anyang, South Korea), and the confluence was determined using a JuLI Br incubator microscope (NanoEnTek, Guro-gu, South Korea). The cell area was then calculated from the cell number, the confluence, and the area of a single acquired field of view. The single cell area was calculated from the quotient of the area covered by cells and the number of cells divided by the field of view.

The growth kinetic reference values resulted from the initial cell number at the first seeding of the cells, the respective cell number on the day of passage, and the time between two passages.

All experiments were performed within passages 3–12. Technical replicates were performed within the same passenger number. For biological replicates, a new batch of cells was thawed in each case. Furthermore, all experiments were performed with *n* ≥ 3 unless otherwise stated. Arithmetical means, standard deviation, and standard errors were calculated using Microsoft Office Excel 2010. Unless otherwise described, the standard error was given. For statistical analysis, outliers were first adjusted using the modified Thompson tau test, the sample was then tested for normal distribution using the Shapiro–Wilk test, and statistical significance was calculated using two-tailed Student’s *t*-tests. Analysis of the confluence data was performed using the analysis of variance test (ANOVA). In the results section, the significances were given as the probability (*p*) of accepting the null hypothesis as true. The significance threshold was assumed to be *p* = 2 ≤ 0.05 (*, *p* < 0.05; **, *p* < 0.01; ***, *p* < 0.001; ns, *p* > 0.05). Statistical analyses and graph plotting were performed by Microsoft Office Excel 2010 and Sigma Plot 12, respectively.

## 3. Results and Discussion

### 3.1. SH-SY5Y Cells Show Distinctive Cell Subtypes

**Figure 1:**
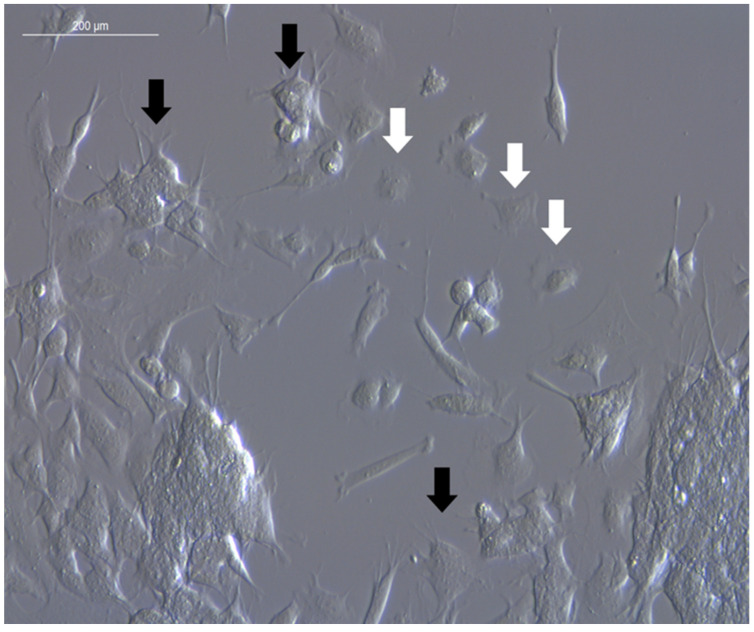
Morphology of SH-SY5Y cells after incubation of 96 h under standard culture conditions. The black arrows mark the neuron-like cells of the N-type; the white arrows point to the epithelial cell-like S-type. The N-type cells have distinctive unpolarized fine cell processes. 200×, IMC.

**Figure 2:**
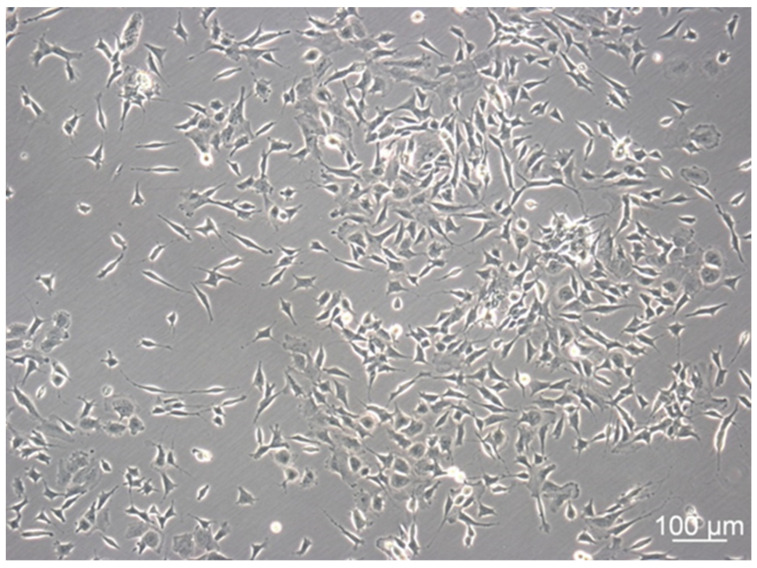
Morphology of SH-SY5Y cells after incubation of 120 h under standard cultivation conditions and an initial cell count of 5 × 10^4^ cells/cm^2^. 100×, PH.

Amidst the adherent cells cultivated in the undifferentiated state, two phenotypes could be distinguished (Figure 1 and Figure 2). Adherent SH-SY5Y cells either depicted the neuron-like N-type or the epithelial-cell-like S-type [29,30]. The N-type showed a similar morphology as fibroblasts with optically sharply defined contours and appeared more elevated from the surface than the S-type. N-Type SH-SY5Y cells formed neurites (fine cell processes), some of which sprouted in a larger number per cell [31]. A major fraction of the cells showed a bipolar morphology with two processes sprouting from opposite ends of the cell body. The S-type was clearly distinguishable as epithelial cell-like. It was less sharply defined, and the cells were flat with the typically “fried egg” form often featured in cultured epithelial cells. S-type SH-SY5Y cells did not develop any extensions and were therefore easy to distinguish from N-type cells.

The transition between S- and N-phenotypes occurred spontaneously, even without the addition of additives or differentiation factors [29]. N-type cells depicted the characteristic morphology of neuroblasts with the capability to differentiate into neuron-like cells. N-type differentiation is achieved by adding the brain-derived neurotrophic factor (BDNF), tumor promoter 12-*O*-tetradecanoylphorbol-13-acetate (TPA), or retinoic acid (RA), which is most commonly used [32]. An overview of the use of various substances to introduce differentiation is described elsewhere [33].

The differentiated N-type cells form neurites (fine cell processes) but do not polarize. Thus, dendrites or axons cannot be discriminated [34]. Furthermore, differentiated N-type SH-SY5Y cells do not form synaptic structures, and neurite projection connections to their neighbors are rare [35]. Therefore, the SH-SY5Y cell line represents a powerful tool for research questions focusing on neuronal outgrowth and tumor progression, maturation, network, or functional studies. Following differentiation, the cells irreversibly enter the G_0_ phase in the cell cycle lacking further cell division, and thus, the assessment of the proliferation rate is precluded.

### 3.2. Verification of Experimental Comparability between Different Culture Samples

**Figure 3:**
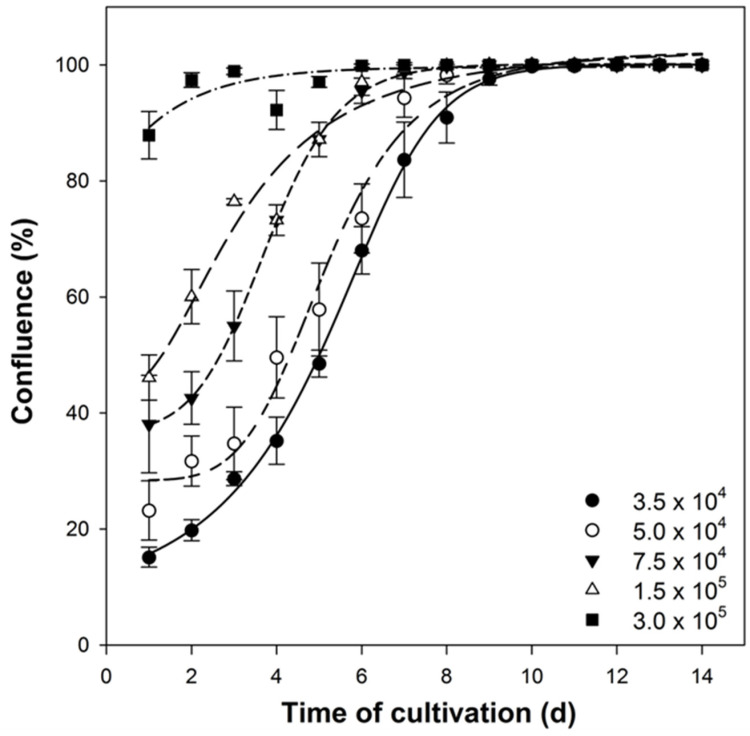
Confluence of SH-SY5Y over a cultivation period of 14 days. Cells were seeded with initial densities of 3.0 × 10^5^ cells/cm^2^, 1.5 × 10^5^ cells/cm^2^, 7.5 × 10^4^ cells/cm^2^, 5.0 × 10^4^ cells/cm^2^, and 3.5 × 10^4^ cells/cm^2^. Confluence was analyzed by bright field live-cell imaging with a microscope stationary within the incubator. (Regression coefficient for the curve adaptions for 3.0 × 10^5^ cells/cm^2^: r^2^ = 0.9721; for 1.5 × 10^5^ cells/cm^2^: r^2^ = 0.9701; for 7.5 × 10^4^ cells/cm^2^: r^2^ = 0.9811; for 5.0 × 10^4^ cells/cm^2^: r^2^ = 0.9881; and for 3.5 × 10^4^ cells/cm^2^: r^2^ = 0.9913).

**Figure 4:**
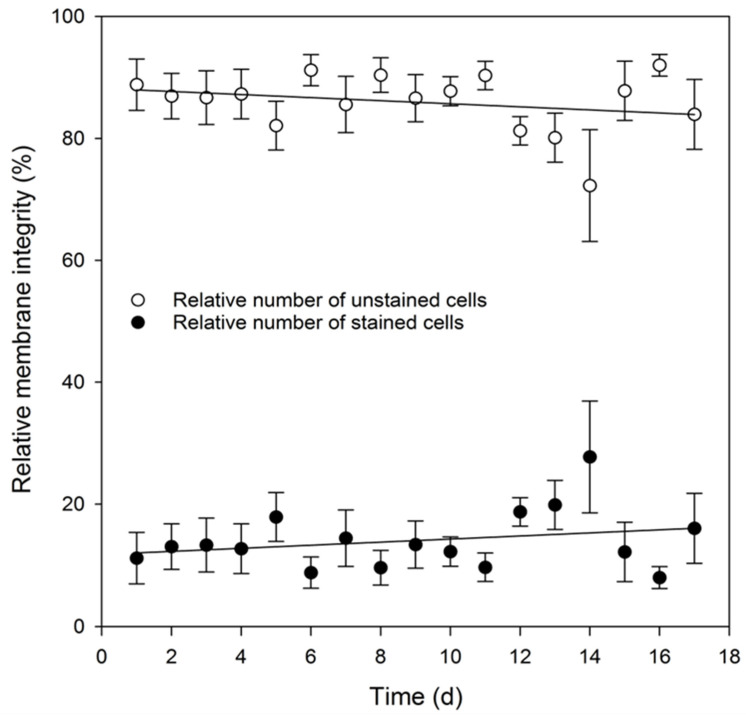
Relative membrane integrity of SH-SY5Y cells over an incubation period of 17 days using the dye permeabilization of trypan blue as a measure. After detaching the cells with trypsin, cells were stained with 0.4% trypan blue, and the number of stained and unstained cells was determined using an automated cell counter. Regression coefficient of the curve adaptations: r^2^ = 0.933.

In order to reliably compare separate experiments with each other, a defined cell number is required that should respond mostly identically to the subsequent culturing conditions. For this, the growth curve of these cells is needed to reveal the first two stages of the lag and log phases. For the SH-SY5Y cell line, the initial cell number of 5.0 × 10^4^ cells/cm^2^ was optimal to achieve an appropriate cell number in an acceptable time frame. For this cell number, the measured growth curve showed a significant delay phase of 3 days, a log phase of 4 days, and a confluence of ~90% after 7 days (Figure 3). This cell number represented an optimal degree of confluency for most types of experiments without having a negative impact on cell integrity (Figure 4). A higher seeding cell number of 7.5 × 10^4^ cells/cm^2^ was possible, but ultimately not profitable, because the time gain was only marginal, featuring an incomplete lag phase. With even higher cell numbers, the lag phase was omitted, which means that the comparability of the experiment results could no longer be guaranteed.

### 3.3. Comparison of Different Cultivation Media

**Figure 5:**
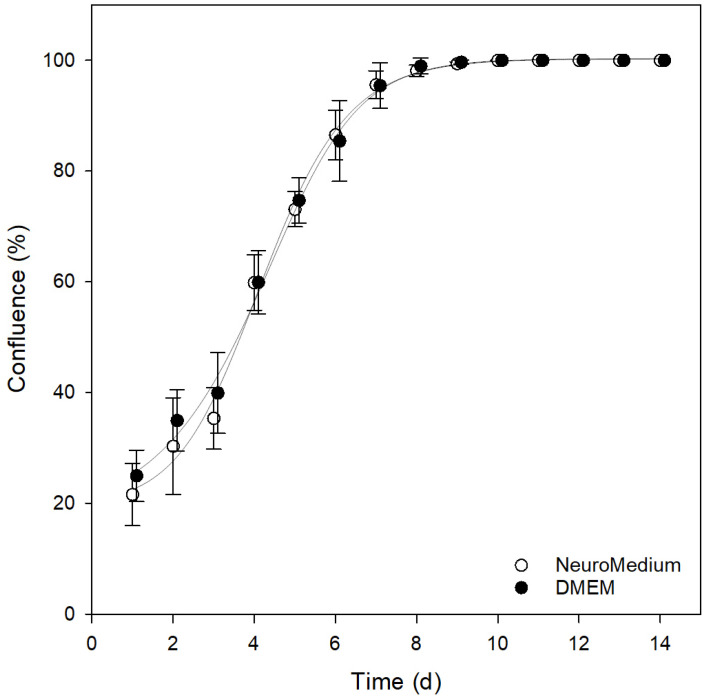
Comparison of SH-SY5Y confluence in the two most commonly used cultivating media during 14 days of cultivation based on the confluence determined by the bright field live-cell imaging. (Regression coefficient for NeuroMedium: r^2^ = 0.9967; for DMEM: r^2^ = 0.9974).

The current literature is inconclusive with regard to an optimal growth-promoting culture medium for SH-SY5Y cells [36,37]. Some groups described a culture of the SH-SY5Y cell line in a 1:1 mixture of DMEM and Ham’s F12; other researchers have described successful culture exclusively in DMEM [31] or RPMI 1640 [29,38]. To resolve this contradiction, we directly compared the growth curves when cultivated in NeuroMedium and DMEM and did not find significant differences (Figure 5). Accordingly, DMEM as a simple medium composition was used in the following experiments. For the differentiation of SH-SY5Y cells, NeuroMedium with Ham’s F12 should be considered due to the requirements of the amino acid and the growth factor composition of SH-SY5Y cells during their differentiation [2].

### 3.4. Analysis of Key Cell Line Characteristics

**Figure 6:**
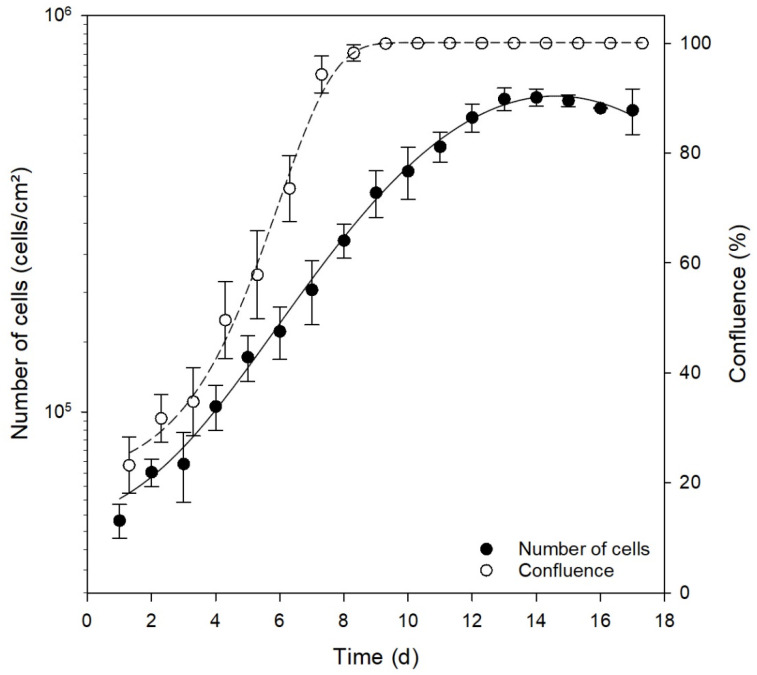
Proliferation of SH-SY5Y based on the cell number and confluence over a period of 17 days. The number of cells and confluence were determined via live-cell imaging within the incubator. (Regression coefficient for total cell number: r^2^ = 0.9956; for confluence: r^2^ = 0.9982).

Growth rate and proliferation rate are the standard values for characterizing a cell line. They are the key features for the division rate and the cell cycle and are thus necessary for every experiment. SH-SY5Y cells proliferated in a typical exponential curve with a saturation character (Figure 6). After a short lag phase of 1 day, SH-SY5Y cells entered the exponential phase at an initial cell count of 5.0 × 10^4^ cells/cm^2^ on day 4 after seeding and finished on day 9 with the contact-induced transition into the stationary phase (Figure 6). During the last stage of the exponential phase, the maximal division rate was observed simultaneously with an increase in confluence to ~98% after 8 days. The log phase lasted until day 12. Cell growth then entered the stationary phase, while confluence remained stable at ~100%. After about 17 days, a maximal cell density of 5.77 × 10^5^ ± 7.61 × 10^4^ cells/cm^2^ was reached, followed by a reduction in cell numbers due to an excessive cell density and thus an induction of cell death (Figure 6).

The viability of the cells can only be determined indirectly. Membrane integrity as viability marker was determined by the permeability for the dye trypan blue [36,37]. A high cell viability is essential to perform reproducible experiments. Values concerning the proliferation rate of SH-SY5Y cells were determined using a Luna automated cell counter (Logos Biosystems, Korea). For each measurement, the cells were trypsinized, dissociated, and resuspended in NeuroMedium as described above. Then a solution containing 0.4% of the dye trypan blue was added to the cell suspension in a 1:1 ratio. This allowed dead cells to be distinguished from living cells by their respective membrane integrity as measured by the permeability of cells towards the trypan blue dye. The stained cells were counted automatically by the cell counting system, and the data were statistically analyzed (Figure 4). For each viability and cell count measurement, ~1.3 × 10^3^ cells were analyzed. The fraction of cells found to be single cells during cell counting and seeding was >98% for all experiments. The remaining 2% that occurred as cell clumps with two or more cells were not considered for further calculations.

The confluence describes the ratio between cell count and cell area, accordingly the relative total area covered by all cells in a culture vessel. At a confluence of 100%, the complete area of the culture vessel is covered by cells. Compared with confluence, the increase in cell number is delayed. Hence, at a confluence of 100%, the cell proliferation progresses even further and only reaches the lag phase with some delay. As experiments should always be initiated in the exponential growth phase, it is important to know the exact confluence curve of the cell line. In this study, the confluence was measured with a microscope located within the cell culture incubator, the so-called live-cell analyzer JuLI Br (NanoEnTek, Korea). To determine the confluence of an individual culture, the culture vessels were imaged at five different positions, followed by averaging and statistical analysis. For each measurement, ~1.7 × 10^4^ cells were analyzed. The calculation of the individual cell area was based on the total cell number of the sample, the total area of the culture vessel, and the field of view at fixed microscope settings that were identical for each experiment.

**Figure 7:**
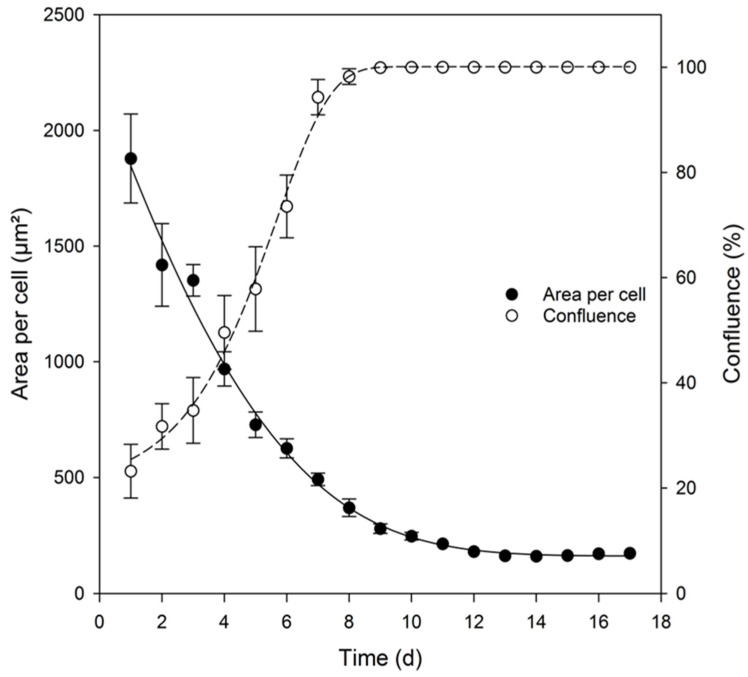
Relationship between confluence and the area of an SH-SY5Y cell over a cultivation period of 17 days. The cell area was determined by the bright field live-cell analyzer JuLI Br. (Regression coefficient for confluency: r^2^ = 0.9982; for the cell area: r^2^ = 0.9848).

The cells showed a confluence-dependent proliferation rate (Figure 6). The cell layer was completely confluent as early as day 8 in culture, but at the same time, the cells were still in the exponential growth phase. A comparison of the entry into the stationary phase of the cell count with confluence showed that the stationary phase was reached 6 days earlier in the case of confluence. This phenomenon could be explained by the comparison between the confluence and the individual cell size (Figure 7). Upon an increase in cell number and thus an increase in confluence, the cell size decreased accordingly (Figure 7). SH-SY5Y cells with an initial cell count of 5.0 × 10^4^ cells/cm^2^ occupied an area per cell corresponding to 1878 ± 192 μm^2^ (~43 × 43 µm). After 17 days in culture, the cell size decreased to 173 ± 13 μm^2^ (~13 × 13 µm). Thus, the cell size was reduced approximately by a factor of 10. Notably, the cells were still capable of dividing, even after full confluence of the cell layer was reached. SH-SY5Y cells are therefore ideal to produce high cell mass (e.g., for biochemical analyses), but the level of confluence needs to be accurately determined to ensure optimal differentiation.

The important parameters for any cell lines are the cell’s doubling time as a reliable measure of the cell line′s proliferation rate over time and the corresponding cell passages. The plating efficiency (PE), the clonogenic dividing ability of a cell line, is low in comparison with other tumor cell lines. The colony-forming capacity is 0.022 ± 0.007. This value is the basis for performing colony-forming assays (CFAs). The use of a conditioned medium at a ratio of 1:10 with a fresh medium increased the PE approximately twice to 0.045 ± 0.012. For this purpose, the medium was removed from a 70% confluent SH-SY5Y culture, supplemented with 10% of fresh NeuroMedium, and filtered sterilely. Growth factors from the extracted and diluted medium of already cultivated cells (conditioned medium) promote an increase in cell growth. This principle of using a conditioned medium is often employed in primary neuronal cell cultures that require the support of glial cells. The neuroblastoma SH-SY5Y cell line, thus, displays similar features in this regard.

The doubling time of SH-SY5Y cells during the exponential phase was 67.3 h ± 5.8 h (Figure 6). In the literature, only inconsistent experimental data on the doubling time of SH-SY5Y were found. Here, the values ranged between 27 h [1] and 55 h [39]. Neither the information provided by the suppliers nor the literature could be confirmed experimentally in over 2 months (63 days) of culture. In our hands, the differences were not due to the cell culture material or the culture medium, since the growth rate of the cultures supplemented with DMEM or NeuroMedium was shown to be very similar (Figure 5).

**Figure 8:**
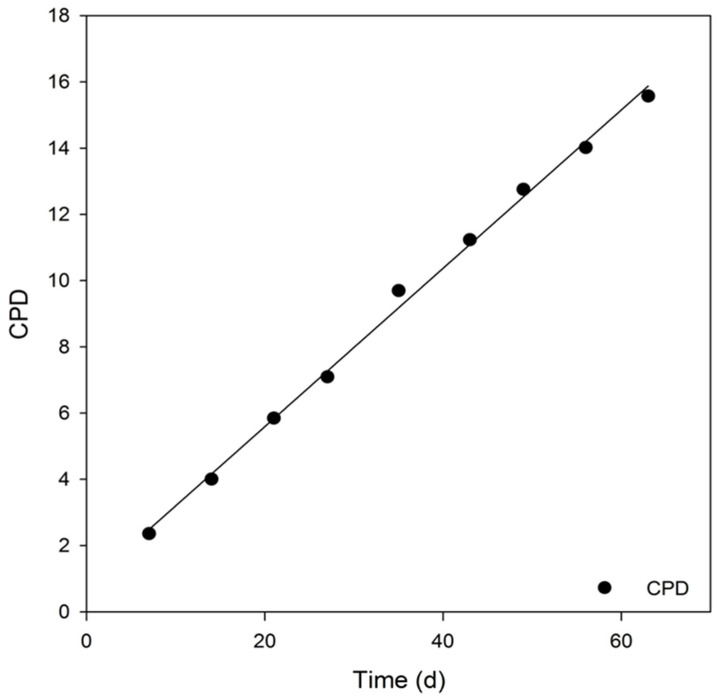
Cumulative population doubling (CPD) of SH-SY5Y over a cultivation period of 63 days. (Regression coefficient: r^2^ = 0.9969).

In 2 months of continuous cultivation, growth kinetic data were derived from the initial cell density (N_0_) and harvested cell density at defined time points (N) as well as the duration of cell growth between two passages. The average generation time (G) was 4.23 ± 0.84 d, the division rate per cell unit (r) was 0.25 ± 0.05, and the multiplication factor (V) was 3.49 ± 1.21. The cumulative population doubling (CPD) after the first passage was 2.36 and increased linearly throughout time (Figure 8). After a culture period of 12 passages and 9 weeks, the CPD amounted to 15.58. Therefore, SH-SY5Y cells showed growth-related parameters that were typical or even superior to many described immortalized tumor-derived cell lines [38]. A decrease in proliferation capacity after 10 passages, as described in the literature, could not be confirmed [40].

## 4. Conclusions

The methods and instructions described here for the cultivation of SH-SY5Y are necessary to achieve perfect conditions for functional studies.

In summary, SH-SY5Y cells can be cultivated at ideal conditions to optimize cell growth phases and cell densities and prevent excessive cell death when considering the culture conditions that were described in this study. It is therefore particularly important to pay attention to the correct initial cell seeding density, as low cell numbers can lead to a slow growth progression or even a complete growth arrest. Regular exchanges of specific culture media are required every 3 days, maintaining high cell viability even after long culture periods of over 2 weeks.

The cumulative population doubling is stable over a longer period of time, which makes a long-term culture and experiments with higher passage numbers possible without any problems. Taking optimal cultivation conditions into account will enable a high degree of reproducibility. For proliferation experiments, one should consider the growth rate relative to confluence, as well as the low colony-forming ability.

## Data Availability

Data are contained within the article in the graphs and in the text.

## References

[1] Kovalevich J., Langford D. (2013). Considerations for the use of SH-SY5Y neuroblastoma cells in neurobiology. Neuronal Cell Culture.

[2] Shipley M.M., Mangold C.A., Szpara M.L. (2016). Differentiation of the SH-SY5Y Human Neuroblastoma Cell Line. J. Vis. Exp..

[3] Um M., Lodish H.F. (2006). Antiapoptotic effects of erythropoietin in differentiated neuroblastoma SH-SY5Y cells require activation of both the STAT5 and AKT signaling pathways. J. Biol. Chem..

[4] Biedler J.L., Helson L., Spengler B.A. (1973). Morphology and growth, tumorigenicity, and cytogenetics of human neuroblastoma cells in continuous culture. Cancer Res..

[5] Biedler J.L., Roffler-Tarlov S., Schachner M., Freedman L.S. (1978). Multiple neurotransmitter synthesis by human neuroblastoma cell lines and clones. Cancer Res..

[6] Ross R.A., Spengler B.A., Biedler J.L. (1983). Coordinate morphological and biochemical interconversion of human neuroblastoma cells. J. Natl. Cancer Inst..

[7] Lopes F.M., Schröder R., da Frota M.L.C., Zanotto-Filho A., Müller C.B., Pires A.S., Meurer R.T., Colpo G.D., Gelain D.P., Kapczinski F. (2010). Comparison between proliferative and neuron-like SH-SY5Y cells as an in vitro model for Parkinson disease studies. Brain Res..

[8] Xicoy H., Wieringa B., Martens G.J.M. (2017). The SH-SY5Y cell line in Parkinson’s disease research: A systematic review. Mol. Neurodegener..

[9] Vaughan P.F., Peers C., Walker J.H. (1995). The use of the human neuroblastoma SH-SY5Y to study the effect of second messengers on noradrenaline release. Gen. Pharmacol. Vasc. Syst..

[10] Khwanraj K., Phruksaniyom C., Madlah S., Dharmasaroja P. (2015). Differential Expression of Tyrosine Hydroxylase Protein and Apoptosis-Related Genes in Differentiated and Undifferentiated SH-SY5Y Neuroblastoma Cells Treated with MPP^+^. Neurol. Res. Int..

[11] Oyarce A.M., Fleming P.J. (1991). Multiple forms of human dopamine beta-hydroxylase in SH-SY5Y neuroblastoma cells. Arch. Biochem. Biophys..

[12] Oe T., Sasayama T., Nagashima T., Muramoto M., Yamazaki T., Morikawa N., Okitsu O., Nishimura S., Aoki T., Katayama Y. (2005). Differences in gene expression profile among SH-SY5Y neuroblastoma subclones with different neurite outgrowth responses to nerve growth factor. J. Neurochem..

[13] Hashemi S.H., Li J.-Y., Ahlman H., Dahlström A. (2003). SSR2(a) receptor expression and adrenergic/cholinergic characteristics in differentiated SH-SY5Y cells. Neurochem. Res..

[14] Deng H., Jankovic J., Guo Y., Xie W., Le W. (2005). Small interfering RNA targeting the PINK1 induces apoptosis in dopaminergic cells SH-SY5Y. Biochem. Biophys. Res. Commun..

[15] Balasubramanian P.S. (2021). Characterization of SH-SY5Y Neurons Subject to 92kHz Ultrasound Stimulation. Int. J. Morphol..

[16] Cohen M.A., Zhang S., Sengupta S., Ma H., Bell G.W., Horton B., Sharma B., George R.E., Spranger S., Jaenisch R. (2020). Formation of human neuroblastoma in mouse-human neural crest chimeras. Cell Stem Cell.

[17] Hu X., Guo X., Ni J., Wang H., Cao N., Liang Z., Wang X. (2020). High homocysteine promotes telomere dysfunction and chromosomal instability in human neuroblastoma SH-SY5Y cells. Mutat. Res. Toxicol. Environ. Mutagen..

[18] Peng Y., Chu S., Yang Y., Zhang Z., Pang Z., Chen N. (2021). Neuroinflammatory In Vitro Cell Culture Models and the Potential Applications for Neurological Disorders. Front. Pharmacol..

[19] Dwane S., Durack E., Kiely P.A. (2013). Optimising parameters for the differentiation of SH-SY5Y cells to study cell adhesion and cell migration. BMC Res. Notes.

[20] Baboo J., Kilbride P., Delahaye M., Milne S., Fonseca F., Blanco M., Meneghel J., Nancekievill A., Gaddum N., Morris G.J. (2019). The Impact of Varying Cooling and Thawing Rates on the Quality of Cryopreserved Human Peripheral Blood T Cells. Sci. Rep..

[21] Mazur P. (1984). Freezing of living cells: Mechanisms and implications. Am. J. Physiol. Physiol..

[22] Galvao J., Davis B., Tilley M., Normando E., Duchen M.R., Cordeiro M.F. (2013). Unexpected low-dose toxicity of the universal solvent DMSO. FASEB J..

[23] Yang W., Tiffany-Castiglioni E., Koh H.C., Son I.-H. (2009). Paraquat activates the IRE1/ASK1/JNK cascade associated with apoptosis in human neuroblastoma SH-SY5Y cells. Toxicol. Lett..

[24] Puck T.T., Marcus P.I., Cieciura S.J. (1956). Clonal growth of mammalian cells in vitro: Growth characteristics of colonies from single HeLa cells with and without a" feeder" layer. J. Exp. Med..

[25] Curtis A.S., Forrester J.V., McInnes C.R., Lawrie F. (1983). Adhesion of cells to polystyrene surfaces. J. Cell Biol..

[26] Constantinescu R., Constantinescu A.T., Reichmann H., Janetzky B. (2007). Neuronal differentiation and long-term culture of the human neuroblastoma line SH-SY5Y. Neuropsychiatric Disorders an Integrative Approach.

[27] Masters J.R., Stacey G.N. (2007). Changing medium and passaging cell lines. Nat. Protoc..

[28] Huang H.-L., Hsing H.-W., Lai T.-C., Chen Y.-W., Lee T.-R., Chan H.-T., Lyu P.-C., Wu C.-L., Lu Y.-C., Lin S.-T. (2010). Trypsin-induced proteome alteration during cell subculture in mammalian cells. J. Biomed. Sci..

[29] La Quaglia M.P., Manchester K.M. (1996). A comparative analysis of neuroblastic and substrate-adherent human neuroblastoma cell lines. J. Pediatr. Surg..

[30] Bell N. (2013). Store-operated Ca^2+^ entry in proliferating and retinoic acid-differentiated N-and S-type neuroblastoma cells. Biochim. Et Biophys. Acta (BBA)-Mol. Cell Res..

[31] Encinas M., Iglesias M., Liu Y., Wang H., Muhaisen A., Ceña V., Gallego C., Comella J.X. (2002). Sequential Treatment of SH-SY5Y Cells with Retinoic Acid and Brain-Derived Neurotrophic Factor Gives Rise to Fully Differentiated, Neurotrophic Factor-Dependent, Human Neuron-Like Cells. J. Neurochem..

[32] Bouché M., Senni M., Grossi A., Zappelli F., Polimeni M., Arnold H., Cossu G., Molinaro M. (1993). TPA-Induced Differentiation of Human Rhabdomyosarcoma Cells: Expression of the Myogenic Regulatory Factors. Exp. Cell Res..

[33] Agholme L., Lindström T., Kågedal K., Marcusson J., Hallbeck M. (2010). An In Vitro Model for Neuroscience: Differentiation of SH-SY5Y Cells into Cells with Morphological and Biochemical Characteristics of Mature Neurons. J. Alzheimer’s Dis..

[34] Ferrari-Toninelli G., Paccioretti S., Francisconi S., Uberti D., Memo M. (2004). TorsinA negatively controls neurite outgrowth of SH-SY5Y human neuronal cell line. Brain Res..

[35] Forster J.I., Köglsberger S., Trefois C., Boyd O., Baumuratov A.S., Buck L., Balling R., Antony P.M.A. (2016). Characterization of Differentiated SH-SY5Y as Neuronal Screening Model Reveals Increased Oxidative Vulnerability. SLAS Discov. Adv. Sci. Drug Discov..

[36] Korecka J.A., van Kesteren R., Blaas E., Spitzer S.O., Kamstra J., Smit A.B., Swaab D., Verhaagen J., Bossers K. (2013). Phenotypic Characterization of Retinoic Acid Differentiated SH-SY5Y Cells by Transcriptional Profiling. PLoS ONE.

[37] Tanimoto R., Hiraiwa T., Nakai Y., Shindo Y., Oka K., Hiroi N., Funahashi A. (2016). Detection of Temperature Difference in Neuronal Cells. Sci. Rep..

[38] La Monica N., Racaniello V.R. (1989). Differences in replication of attenuated and neurovirulent polioviruses in human neuroblastoma cell line SH-SY5Y. J. Virol..

[39] Um M., Gross A.W., Lodish H.F. (2007). A “classical” homodimeric erythropoietin receptor is essential for the antiapoptotic effects of erythropoietin on differentiated neuroblastoma SH-SY5Y and pheochromocytoma PC-12 cells. Cell. Signal..

[40] Tennant J.R. (1964). Evaluation of the trypan blue technique for determination of cell viability. Transplantation.

